# Spatial Distribution, Sources, Air–Soil Exchange, and Health Risks of Parent PAHs and Derivative-Alkylated PAHs in Different Functional Areas of an Oilfield Area in the Yellow River Delta, North China

**DOI:** 10.3390/toxics11060540

**Published:** 2023-06-17

**Authors:** Xiongfei Zhang, Anan Qi, Pengcheng Wang, Qi Huang, Tong Zhao, Caiqing Yan, Lingxiao Yang, Wenxing Wang

**Affiliations:** 1 Environment Research Institute, Shandong University, Qingdao 266237, China; zhangxiongfeisddx@163.com (X.Z.); ananqi101@163.com (A.Q.); 17865310973@163.com (P.W.); huangqi2017@163.com (Q.H.); zhaotong8213@163.com (T.Z.); cyan0325@sdu.edu.cn (C.Y.); wxwang@sdu.edu.cn (W.W.); 2 Jiangsu Collaborative Innovation Center for Climate Change, Nanjing 210093, China

**Keywords:** oilfield, polycyclic aromatic compounds, PMF, air–soil exchange, health risk assessment

## Abstract

The knowledge of the spatial distribution, sources, and air–soil exchange of polycyclic aromatic compounds (PACs) in an oilfield area is essential to the development of effective control practices of PAC pollution. In this study, 48 passive air samples and 24 soil samples were collected during 2018–2019 in seven functional areas (e.g., urban, oil field, suburban, industrial, agricultural, near pump units, and background) in the Yellow River Delta (YRD) where the Shengli Oilfield is located, and 18 parent polycyclic aromatic hydrocarbons (PAHs) and five alkylated-PAHs (APAHs) were analyzed from all the air and soil samples. The ΣPAHs in the air and soil ranged from 2.26 to 135.83 ng/m^3^ and 33.96 to 408.94 ng/g, while the ΣAPAHs in the atmosphere and soil ranged from 0.04 to 16.31 ng/m^3^ and 6.39 to 211.86 ng/g, respectively. There was a downward trend of atmospheric ΣPAH concentrations with increasing the distance from the urban area, while both ΣPAH and ΣAPAH concentrations in the soil decreased with distance from the oilfield area. PMF analyses show that for atmospheric PACs, coal/biomass combustion was the main contributor in urban, suburban, and agricultural areas, while crude production and processing source contributes more in the industrial and oilfield area. For PACs in soil, densely populated areas (industrial, urban, and suburban) are more affected by traffic sources, while oilfield and near-pump unit areas are under the impact of oil spills. The fugacity fraction ($f_{f}$) results indicated that the soil generally emitted low-molecular-weight PAHs and APAHs and act as a sink for high-molecular-weight PAHs. The incremental lifetime cancer risk (ILCR) of Σ(PAH+APAH) in both the air and soil, were below the threshold (≤10^−6^) set by the US EPA.

## 1. Introduction

The environmental pollution caused by oil extraction and utilization has become a major environmental issue [1,2,3]. Many polycyclic aromatic compounds (PACs) are released by oil extraction activities into the environment, including polycyclic aromatic hydrocarbons (PAHs) and alkylated PAHs (APAHs) [4,5,6]. Although the proportion of PAHs in crude oil is not high (less than 2% in weight) [7], long-term oil extraction leads to the accumulation of high PAH concentrations in nearby environmental media, such as soil [5] and the atmosphere [8]. APAHs are the representative substance in crude oil, the concentration of APAHs in crude oil is much higher than the parent PAHs, and higher concentration of APAHs were also found in the atmosphere around oil sand areas [6]. Some APAHs are more toxic than parent PAHs and deserve special attention [8,9].

In the world’s largest oil sand area in Northern Alberta, Canada, a series of measurements have been conducted to study the impact of oil sand mining on PAC contamination in the local atmosphere and soil [8,10,11]. Stack emissions, fugitive emissions from mining activities, and heavy transport activities from oil sand operations were reported to be the main source of atmospheric PAHs [8,10]. Additionally, the concentration of atmospheric PAHs and APAHs declined exponentially within 50 km from the central of oil sand area [6,8], indicating that PAHs and APAHs in oil sand area are mainly related with oil sand mining activities. Soil sampling and analysis showed that PAH pollution from the oil sand area can travel long distances and even pollute remote areas [4], confirming that oil sand extraction has a considerable impact on PAH and APAH loading in a wide range of areas [12].

However, there are notable differences between the extraction processes in oil sand areas and oilfields [13,14,15]. A study in the Shengli oilfield reported that the health risk caused by PAHs in the surrounding soil of an oil well rose with increasing oil well extraction time [5]. The soil from several oilfields [2] revealed the proportion of lower molecular weight PAHs in the soil near the oil well was relatively high, which indicates oil leakage of ground crude oil. In addition to the direct pollution from oil extraction, soils around an oilfield area are also vulnerable to PAHs emitted by heavy transportation [5,16]. These findings were mainly about soil samples [7,17,18] since the soil has long been considered a sink for PACs. However, Ma, et al. [19] found that persistent organic pollutants previously stored in the soil can be recycled into the environment due to the impact of global warming. Keyte, et al. [20] also suggested that PACs will revolatilize into the atmosphere when temperatures rise. In recent years, investigations into the air-soil exchange of PAHs demonstrated that soil is an important source of atmospheric PAHs [21,22]. Hsu, et al. [10] found that concentrations of some PAHs (mainly 3–4 rings) in the atmosphere in oil sand areas varied seasonally with temperature, suggesting this was due to the exchange of PAHs between the atmosphere and soil. This factor will also affect the travel distance and atmospheric lifetime of PACs in oil development areas [8]. Compared with the parent PAHs, the research on APAHs in oilfield areas was limited. Therefore, the study of the air–soil exchange of PAHs and APAHs is essential for understanding the fate and lifecycle of PAHs and APAHs (for example, secondary emission and transfer of PAHs and APAHs in different functional areas) and pollution control in oilfield areas.

According to the BP Statistical Review of World Energy 2021, 70th edition, China is the sixth largest producer of oil, with 3901 thousand barrels per day. The Shengli oilfield is one of the largest oilfields in China, with an oil production of 23.4 million tons in 2022, and has been operating for more than 50 years. Its eastern main production area is situated in the Yellow River Delta (YRD). Paralleling the development of the Shengli Oilfield, the industrial areas and port traffic in the YRD have also been expanding. The surrounding areas have been gradually urbanized, and there are now millions of people living in this area [5]. The overlap of long-term crude oil extraction and high population density might lead to significant health consequences [8,23]. Wu, et al. [7] revealed that if no effective measures or policies were taken, the health risk caused by exposure to PAHs in the soil in YRD would exceed the threshold of 10^−6^ (set by the US EPA) around 2025, posing a threat to the health of residents.

In previous studies in the YRD region, researchers were more concerned about parent PAHs in the soil [7,16,17,24], and only few involved atmospheric PAHs [25]. In addition, research on the migration of atmospheric and soil PAHs in oil field extraction areas is limited. In this research, we systematically study the pollution characteristics, environmental behavior, concentration levels, and the impact of oil field exploitation on PAH and APAH in the atmosphere and soil of the oil field region. Additionally, the trend of air-soil exchange in the region was also investigated, as well as the fate of PAH and APAH in the soil and atmosphere during different seasons.

In this study, 48 atmospheric samples and 24 topsoil samples were collected from 2018 to 2019, and the concentrations of 18 PAHs and 5 APAHs were determined. The objectives of this research include: (1) to measure the concentrations and investigate the spatial distribution of PAHs and APAHs in the ambient air and soil in the YRD region, (2) to assess possible sources of PAHs and APAHs in air and soil of the YRD area, (3) to evaluate the air–soil exchange status of PAHs and APAHs, and (4) to estimate the potential cancer risk of PAHs and APAHs in the YRD region.

## 2. Experimental Section

### 2.1. Sampling Site

The Yellow River Delta (YRD, 36°55′~38°10′ N, 118°07′~119°10′ E) is situated in the northeast of Shandong Province, China, with a total area of about 5400 km^2^. The YRD region is rich in oil resources and is the main oil-producing region of the Shengli oilfield. As a typical traditional oilfield area, oil wells are widely distributed in this area. In order to evaluate the impact of the oil exploitation and processing on PAH and APAH concentrations in different functional areas of the YRD region, the YRD is divided into 7 types of areas, including oil field (O), near-pump unit (P), industrial (I), urban (U), suburban (S), agricultural (A) and background area (B). A total of 24 sampling sites were selected to collect atmospheric and soil samples.

In this study, three oil field sampling sites (O1–O3) were chosen in Gudong oilfield. The Gudong oilfield (annual oil production of 1.2 million tons) is a large integrated drape anticline oilfield near Bohai Bay with an oil bearing area of 55.3 km^2^ and has been in production for more than 30 years. The oil wells are densely distributed here (1818 oilwells in 2020), and oil tank trucks frequently travel between the oil fields and industrial areas. There are two towns (Gudao town and Xianhe town) located 15–25 km west of Gudong Oilfield. Gudao town and Xianhe town are surrounded by many isolated beam pump units and both of which have a population of about 36,000. To better understand the impact of crude oil exploitation and extraction on the health risks of nearby residents, four beam pump units site (P1–P4) in the adjacent residential area as well as four suburban sites (S1–S4) in residential area of Gudao Town and Xianhe town were chosen. In the YRD, the industrial zones are mainly composed of petrochemical industries, with a crude oil processing capacity of more than 15.8 million tons. Five sampling points (I1–I5) were chosen for PAH and APAH observation in these industrial zones. Three urban sites (U1–U3) were selected in the downtown area of Hekou District, a district of Dongying City on the northeast side of the Yellow River Estuary, which has a population of about 200,000 in 2020. Three agricultural sampling sites (A1–A3) were located in the farmland and forest land around Huanghekou town, an agricultural town with more than 39,300 hectares of economic plants, grain, and oil crops, with most of the local population engaging in agriculture. Additionally, a suburban sampling point (S5) was set inside of Huanghekou Town. The background points (B) were at the YRD coastal wetland ecological test station operated by the Chinese Academy of Sciences near the Yellow River Nature Reserve. The region is sparsely populated and far from most anthropogenic pollution sources. All the sampling points were 50 m away from the highway. Detailed information on the 24 sampling sites are shown in Table S1.

### 2.2. Sample Collection

#### 2.2.1. Passive Air Sampling

Passive samplers with polyurethane foam (PUF) disks were used to collect atmospheric samples. Samplers were purchased from Beijing Convenient Environmental Tech Co., Ltd. (Beijing, China). The PUF disks (TE-1040, Tisch Environmental, Inc., Cleves, OH, USA) were stored inside the sampler and kept suspended using two screws.

**Figure 1:**
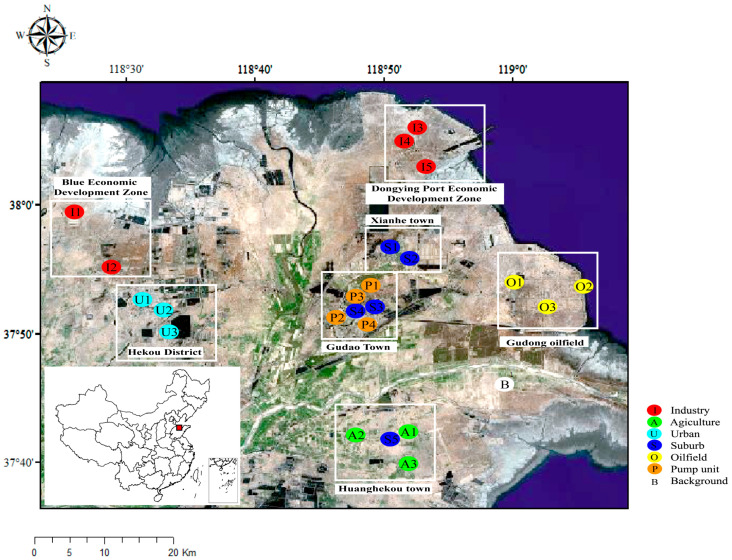
Location and functional area classification of the 24 sampling sites in the YRD area. (Note: In this study, air and soil samples were collected at the same sites.)

Before sampling, PUF disks were purified by Soxhlet extraction 150 mL dichloromethane (DCM) for 8 h. Then, the purified PUF was wrapped in a clean sealed bag. PUF disks were deployed at 24 different sites during the periods from October to December 2018 (60 days, wintertime) and from May to July 2019 (60 days, summertime), respectively. At all the sampling sites, the passive sampler was placed 3 m above the ground. The sampling sites are shown in Figure 1.

#### 2.2.2. Soil Sampling

In 24 December 2018 topsoil samples (0–20 cm) were collected at the same sites as the atmospheric sampling points. At each sampling site, 5 subsamples (collected from an area of 100 square meters) were collected by a stainless steel shovel and mixed into one composite sample. The soil samples were stored at −20 °C until extracted.

### 2.3. Extraction and Analysis

Soxhlet extraction was used in this study. For atmospheric samples, the whole PUF was added to the stripping tube. For the soil samples, 10 g of the soil sample (freeze-dried, ground, and then screened with 200 mess stainless steel sieves) was mixed with 10 g of anhydrous sodium sulfate, added to a casing, and then put into the stripping tube. Detailed information for the extraction can be found in our previous studies [26].

Briefly, the samples were extracted by Soxhlet extraction with 150 mL of dichloromethane (DCM, J.T. Baker, Morristown, NJ, USA) for 8 h. Next, the extracts were concentrated to 1–2 mL using a rotary evaporator (RE201, Shanghai BingYue Corporation, Shanghai, China) and purified through a silicone and alumina column. The column was eluted with 20 mL of n-hexane (discharged) and 70 mL of n-hexane/DCM (1:1, *v*/*v*). The elution was concentrated to 1–2 mL again using a rotary evaporator, and then 10 mL of n-hexane was added to the solvent. Then, the elution was concentrated to 1–2 mL once again using rotary evaporator and then further concentrated to 0.5 mL under a nitrogen stream. Next, 100 ng of internal standards (naphthalene-d8, anthracene-d10, pyrene-d10, and perylene-d12) were spiked for quantification. Finally, the concentrated solution was diluted to 1ml with n-hexane, and then stored at −20 °C before analysis.

After the extraction, a gas chromatography-mass spectrometry (GC-MS, TRACE1300-ISQ7000) with a GC column (TG-5SILMS, 30 m × 0.25 mm × 0.25 μm, Thermo-Fisher Scientific, USA) was used to quantify the PAC concentrations. Ion scan mode (SIM) and electron impact ion source (EI) were employed during the analysis. The oven temperature program was as follows: 60 °C for 1 min, then increased to 160 °C at the rate of 20 °C, held for 1 min, increased to 280 °C at the rate of 6 °C/min, held for 5 min, raised to 300 °C at the rate of 20 °C/min, then held for 8 min. High-purity helium (≥99.999%) was used as a carrier gas with an inlet temperature of 310 °C at a flow of 1 mL/min.

The quantified 18 PAHs and 5 APAHs and their abbreviation are listed in Table S2. PAHs were grouped into 3 classes: low molecular weight (LMW; 2–3 rings), middle molecular weight (MMW, 4 rings), and high molecular weight (HMW, 5–7 rings), according to the US EPA method [27].

### 2.4. Quality Control

In this study, a strict cleaning procedure was conducted for the passive sampler before sampling. It was washed three times with pure water and ultrapure water and then washed with HPLC-grade ethanol. A certain concentration level of mixing standard of PAHs and APAHs were used to check the stability of the GC-MS every day. In this study, the contents of target compounds detected in lab/field blanks were below the detection limit. Acenaphthene-d10 and chrysene-d12 were randomly added to one-third of the samples before extraction. The surrogate recoveries were 94% ± 21% (acenaphthene-d10) and 74% ± 4% (chrysene-d12) for the atmospheric samples, and 96% ± 16% (acenaphthene-d10) and 104% ± 7% (chrysene-d12) for the soil samples. The method detection limit (MDL) for individual PACs ranged from 0.11 × 10^−2^ to 12.50 × 10^−2^ ng/m^3^ in the atmosphere and 0.02 to 2.00 ng/g in the soil. The MDL for specific PAHs and APAHs can be found in Table S2. Most standard solution used in this research were certified reference materials purchased from AccuStandard^®^ Inc. New Haven, CT, USA, while 1M-NAP and 9M-PHE were purchased from Beijing Tanmo Quality Inspection Technology Co., Ltd., Beijing, China and CATO Research Chemicals Inc. Guangzhou, Guangdong province, China, respectively.

### 2.5. Data Analysis

#### 2.5.1. Passive Air Sampling Rate Calculation

In this study, the sampling volume of specific PAHs for passive air samplers were calculated separately [28,29,30], and the specific calculation method has been reported in our previous research [31].

#### 2.5.2. Estimation of Health Risk

The incremental lifetime cancer risk (ILCR) was adopted to assess the carcinogenicity of PAHs and APAHs in ambient air and soil in this study. The ILCR of the air samples for inhalation risk was determined by the following Equation (1):

(1) $${ILCR}_{air}=\frac{{TEQ}_{air}\times {CSF}_{inhalation}\times {IR}_{inhalation}\times EF\times ED}{BW\times AT}$$

Total BaP-equivalent concentration (TEQ) is the sum of BaP_eq_ derived from a PAC concentration multiplied by its corresponding toxic equivalent factor (TEF) [32]. The TEF values were derived from previous studies [33,34,35,36] and are shown in Table S3. CSF is the carcinogenic slope factor (mg kg^−1^ day^−1^)^−1^, IR_inhalation_ is the inhalation rate (m^3^/day), EF is the exposure frequency (day/year), ED is the lifetime exposure duration (year), BW is body weight (kg), and AT is the averaging lifetime of the carcinogenic substance (day).

The cancer risk for the soil samples was obtained by summing the three individual risks [37] and was calculated using Equations (2)–(5) [38]:

(2) $${ILCR}_{soil}={ILCR}_{soil-ingest}+{ILCR}_{soil-dermal}+ILCR_{soil-inhalation}$$

(3) $${ILCR}_{soil-ingest}={CSF}_{ingest}\times \frac{{TEQ}_{soil}\times \left(\sqrt[{3}]{\frac{BW}{70}}\right)\times {IR}_{ingest}\times EF\times ED}{BW\times AT\times 10^{6}}$$

(4) $${ILCR}_{soil-dermal}={CSF}_{dermal}\times \frac{{TEQ}_{soil}\times \left(\sqrt[{3}]{\frac{BW}{70}}\right)\times SA\times SAF\times ABS\times EF\times ED}{BW\times AT\times 10^{6}}$$

(5) $${ILCR}_{soil-inhalation}={CSF}_{inhalation}\times \frac{{TEQ}_{soil}\times \left(\sqrt[{3}]{\frac{BW}{70}}\right)\times {IR}_{inhalation}\times EF\times ED}{BW\times AT\times PEF}$$

where ILCR_ingest_ is the cancer risk through ingestion, ILCR_dermal_ is the cancer risk through contact, and ILCR_inhalation_ is the cancer risk through inhalation. SA is the skin area (cm^2^/day), SAF is the skin adherence factor (mg/cm^2^), ABS is the dermal absorption factor, and PEF is the particulate emission factor (m^3^/kg) [38]. Detailed parameters can be found in Table S4 [39,40,41,42] as well as in our previous studies [27].

The mutagenicity equivalent (MEQ) was introduced to assess the mutagenic equivalents of PAHs and APAHs [32]. MEQ is the sum of PAC concentration multiplied by its corresponding mutagenicity coefficients and was calculated using Equation (6) [43]:

(6) $$\begin{array}{c} MEQ=0.00056\times \left[ACY\right]+0.082\times \left[BaA\right]+0.017\times \left[CHR\right]+0.25\times \left[BbF\right]\\ \;\;\;\;\;\;+\;0.11\times \left[BkF\right]+0.0017\times \left[BeP\right]+1\times \left[BaP\right]+0.31\times \left[IcdP\right]\\ \;\;\;\;\;\;+\;0.29\times \left[DahA\right]+0.19\times \left[BghiP\right]+0.0025\times \left[1M-NAP\right]\\ \;\;\;\;\;\;\;\;\;\;+0.63\times \left[5M-CHR\right] \end{array}$$

#### 2.5.3. Fugacity Fraction Calculation

The fugacity fraction ($f_{f}$) was used to estimate the air–soil exchange trend of PAHs and APAHs. The fugacity fractions were calculated using the following Equation (7) [22,44]:

(7) $$f_{f}=\frac{f_{s}}{f_{s}+f_{a}}$$

where $f_{s}$ and $f_{a}$ are the PAC fugacity for soil and air, respectively. The air fugacity ($f_{a}$) and soil fugacity ($f_{s}$) were estimated based on the following Equations (8) and (9) [22]:

(8) $$f_{a}=C_{a}RT$$

(9) $$f_{s}=\frac{C_{s}\rho_{s}RT}{0.411\varphi_{om}K_{oa}}$$

where C_a_ is the PAC concentration (ng m^−3^) in ambient air, C_s_ represents the PAC concentration (ng g^−1^) in soil; R is a gas constant (8.314 J mol^−1^ K^−1^); T is the temperature (K); $\rho_{s}$ is the density of the soil (kg m^−3^), for which 1.5 × 10^3^ kg m^−3^ was used for all calculations in this study [45]; $\varphi_{om}$ is the organic carbon fraction; and $K_{oa}$ is the octanol-air partition coefficient [46]. The $K_{oa}$ value used in this research is given in Table S5 [47]. Fugacity fractions ranged from 0.3 to 0.7, indicating an equilibrium in the air–soil exchange of PACs. Fugacity fractions greater than 0.7 signified that the soil was a net volatile source for PACs, and fractions less than 0.3 signified that the soil was a net sink for PACs [22,48].

It should be noted that the $f_{f}$ values calculated in this study are only based on the wintertime soil concentrations, with the assumption that PAH and APAH concentrations in the soil were not highly variable among seasons. This can be supported by other studies conducted in different seasons in the YRD region. For example, the reported value for soil collected in August was 119 ng/g [16], and the reported value in May was 118 ng/g [17].

## 3. Results and Discussion

### 3.1. Overview of PAHs and APAHs Concentration in Air and Soil Samples

The concentrations of the individual PAHs and APAHs in the measured air samples from the 24 sampling sites in the YRD are presented in Figures S1 and S2. The concentration of ΣPAHs in the atmospheric samples ranged from 2.26 ng/m^3^ to 135.83 ng/m^3^ (average 41.61 ng/m^3^) and was dominated by volatile and semivolatile 2–4 ring PAHs (accounting for 88.6–98.7% of ΣPAHs). The concentration level of ΣAPAHs in the atmosphere ranged from 0.04 to 16.31 ng/m^3^ (average 2.31 ng/m^3^). Zhang, et al. [49] reported the concentrations of PAHs in the atmosphere of several provinces in North China, including Shandong (212 ± 19 ng/m^3^), Hebei (192 ± 17 ng/m^3^), and the Beijing–Tianjin area (148 ± 28 ng/m^3^), which were all higher than the ΣPAHs concentrations observed in this study. However, the concentrations of PAHs in this study was higher than that observed in the oil sand area in Alberta, Canada (0.03–210 ng/m^3^, average 12.7 ng/m^3^) [6]. By contrast, the concentration of atmospheric APAHs in this study (0.04 to 16.31 ng/m^3^, average 2.31 ng/m^3^) was significantly lower than that in Canada’s oil sands area (0.15–230 ng/m^3^) [6]. This could be attributed to the fact that the species of APAHs detected in this study were limited, as different emissions of APAHs can be found in oil fields using different operation methods. There are a large number of open pits and pools in Canada’s oil sands area, which is conducive to the entry of APAHs into the atmosphere. In contrast, the oil wells in the Yellow River Delta are transported by oil tank truck and an oil pipeline, and therefore, the leakage path of APAH is reduced.

The ΣPAH and ΣAPAH concentrations in the soil samples ranged from 33.96 to 408.94 ng/g (average 125.79 ng/g) and from 6.39 to 221.86 ng/g (average 35.80 ng/g), respectively. The ΣPAHs concentration was consistent with the result by Yuan, et al. [16] and Yuan, et al. [17] in the Shengli oilfield in the YRD region (119 ng/g and 118 ng/g, respectively). It was higher than the concentration observed in the bitumen extraction site (3.2–666.7 ng/g; average 50.4 ng/g) [50] in Alberta, Canada.

### 3.2. Temporal and Spatial Distribution of PAHs and APAHs in Air and Soil

#### 3.2.1. PAHs and APAHs in the Air

**Figure 2:**
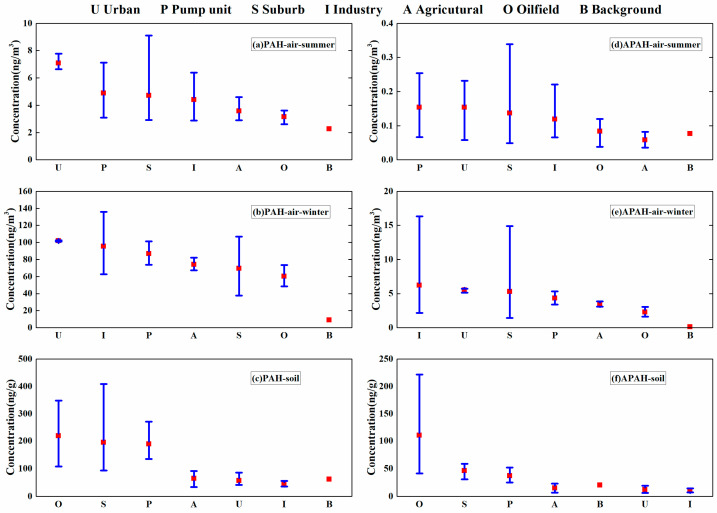
The concentrations of ΣPACs in different functional areas. (**a**) PAHs in summer air (ng/m^3^); (**b**) PAHs in winter air (ng/m^3^); (**c**) PAHs in soil (ng/g); (**d**) APAHs in summer air (ng/m^3^); (**e**) APAHs in winter air (ng/m^3^); (**f**) APAHs in soil (ng/g). Note: The lower and upper limits of the whiskers indicate minimum and maximum, and the small red square represents mean.

**Figure 3:**
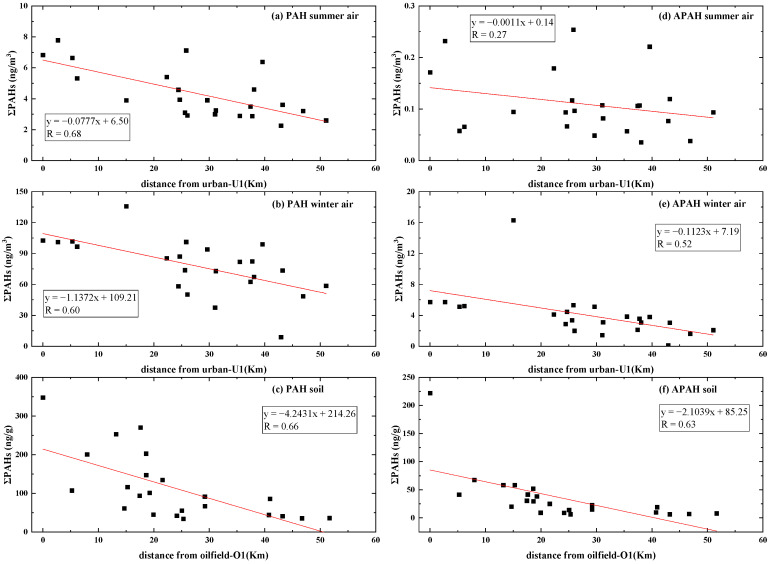
ΣPAH and ΣAPAH concentrations with increasing distance from urban area in air in (**a**,**d**) summer and (**b**,**e**) winter, and ΣPAHs and ΣAPAHs concentrations with increasing distance from oilfield area in soil (**c**,**f**). The red line and the boxes are trend lines and corresponding formulas, respectively. (Note: point S5 was excluded, as there was a market at point S5 every five days which may lead to complex PAH and APAH sources.)

The concentration of ΣPACs from the different functional areas in summer and winter are shown in Figure 2. In this study, the urban area had the highest ΣPAH concentration and a relatively high ΣAPAH concentration in both seasons. In both seasons, the atmospheric ΣPAH concentration exhibited a moderate downward trend with increasing distance from the urban area (U1), which was a densely populated urban sampling site inside the community in the main urban area of Hekou District (Figure 3a,b). However, for APAHs, this trend was only found in winter. For all air samples, PHE was the most common PAH, accounting for 40.3% of ΣPAHs, while 9M-PHE was the most common APAH, accounting for 60.5% of ΣAPAHs. Significant seasonal variations in the ΣPAHs and ΣAPAHs were observed (summer: ΣPAHs 4.53 ± 1.87 ng/m^3^, ΣAPAHs 0.12 ± 0.07 ng/m^3^; winter: ΣPAHs 78.69 ± 27.06 ng/m^3^, ΣAPAHs 4.51 ± 3.65 ng/m^3^; *p* < 0.01). Among all the PAHs, the proportion of FLU increased the most between seasons from 0.42 ng/m^3^ (5.4%) in the summer to 12.1 ng/m^3^ (15.7%) in the winter. FLU, PHE, and FLT accounted for the majority (average 71.7%) of the ΣPAHs in the winter samples, which corresponds to emission profiles of firewood, straw, and coal combustion [51,52]. Methylphenanthrene (9M-PHE and 3,6D-PHE), which might originate from coal and biomass combustion [53], accounted for the majority of the ΣAPAHs in atmosphere (68.0% in summer and 70.4% in winter). Higher ΣPAH and ΣAPAH concentrations were found at site I1 (135.83 ng/m^3^ and 16.31 ng/m^3^, respectively), S5 (106.78 ng/m^3^ and 14.92 ng/m^3^, respectively), U1 (102.55 ng/m^3^ and 5.72 ng/m^3^, respectively), and P4 (101.16 ng/m^3^ and 5.33 ng/m^3^, respectively) during the winter period. These sampling points were located in areas requiring central heating, suggesting that the elevated atmospheric PAC concentrations in winter may be closely related to heating activities in the YRD region [54,55,56]. In this study, the atmospheric ΣPAH concentrations in winter were 12–36 times higher than those in summer, while ΣAPAH concentrations in winter were 13–172 times higher than those in summer (except for the background point). This trend has also been observed by other researchers in North China [31,54,57] and could be attributed to the large amount of PAC emissions in urban areas during heating season [58,59]. Moreover, the unfavorable diffusion conditions that are caused by the lower mixing height in winter and reduced photolytic degradation rate of PACs caused by shorter daylight in the winter intensify the accumulation of PACs in the atmosphere [44,60]. The steeper fitting curve (closer to −1) observed in the winter (Figure 3b,e) indicates the ΣPAH and ΣAPAH concentration both decreases faster with distance in winter compared to in summer and also confirms poor atmospheric diffusion conditions in winter. In the oil sand area in Canada, atmospheric ΣPAH and ΣAPAH concentrations exhibited a moderate downward trend with increasing distance from the oil sand mining region, where an emission inventory indicates stack and heavy transport vehicles are the main emission sources of PAHs [6,10]. This reveals that the contribution of the oil sand area and oil field to the atmospheric PAHs are different. Notably, high proportion of ACE (21.9%–40.7%) were detected in the agricultural (A1–A3) and urban sites (U3) in summer. Previous studies have indicated that ACE is emitted by the volatilization of petroleum products [31], insecticides, and bactericides [61]. Since the A1–A3 sites were in farmland/woodland areas and U3 was in the woods in the community, these results indicate that the agricultural areas and some urban sites were affected by insecticide and bactericide use in summer.

#### 3.2.2. PAHs and APAHs in Soil

The concentrations of the individual PACs in soil at different sampling sites are presented in Figures S1 and S2. Clearly, FLU (14.4%), PHE (14.2%), and CHR (9.4%) were the dominant PAHs measured in the soil samples, while 9M-PHE (37.6%) and 1M-NAP (35.9%) were the dominant APAHs. The high proportion of 1M-NAP indicated that petrogenic sources play an important role in soil of the YRD area especially in oilfield/suburban/pump unit areas [53]. Unlike in the air samples, ΣPAHs concentrations in soil samples taken from areas close to an oil well (oilfield: 218.56 ng/g; suburban: 194.62 ng/g; pump unit: 188.81 ng/g) were significantly higher than those in other areas (*p* < 0.01). Similar to parent PAHs, higher concentration of ΣAPAHs were observed in areas close to an oil well (oilfield: 110.36 ng/g; suburban: 46.34 ng/g; pump unit: 37.10 ng/g). Especially, higher proportions of low–middle molecular weight PAHs (2–4 ring) were observed in the oilfield (69.2%) and suburban (69.2%) areas, particularly for PHE (36.7% of LMW PAHs) and CHR (34.0% of MMW PAHs). PHE and CHR were also the dominant component (37.1%–63.6%) reported by Fu, et al. [5] and Wang, et al. [23] in the soil around oil wells in the Shengli, Daqing, Xinjiang, and Huabei oilfields; however, the proportion of HMW PAHs in Shengli oilfield in this study (30.8%) was higher than in Daqing (1.5%), Xinjiang (0.9%), and Huabei (1.2%) oilfields [23], and a higher proportion of HMW PAHs in Shengli oilfield have also been found by Yuan, et al. [16] and Fu, et al. [5], which may be caused by traffic emission. Zhao, et al. [62] studied the composition of PAHs in crude oil and found that LMW PAH accounted for a large proportion (more than 40%), and PHE was the most abundant LMW PAH in crude oil. LMW PAHs generally originate from low-temperature combustion of organic matter, oil leakage, and ground crude oil [2,17]. Compared with oilfield areas where oil wells are densely distributed, the suburban sampling sites (S1–S4) were mainly located in residential areas in Gudao town and Xianhe town. Higher concentrations of FLT and IcdP, which are generally considered to be indicators of gasoline emissions [26,63,64,65], were found in the residential areas compared to the oilfield areas. FLT is also one of the major products of wood combustion [52], indicating that suburban areas in the YRD were affected by residential combustion and traffic sources. Similar conclusions were drawn from the analysis of topsoil samples in 28 cities in multiple regions in China [66]. Unlike the soil samples from the oilfield sites, the proportion of LMW PAHs near the pump unit sites was relatively low (22.7%). HMW PAHs (5–7 ring) were the dominant PAHs (49.2%). HMW PAHs, such as BeP, IcdP, BghiP, and COR, are indicators of vehicle emission [64,65]. The oil wells near the pump unit sites are not as dense as those in the oil field areas, with 1 to 3 oil wells near each site; therefore, oil tank trucks were used for transporting the crude oil produced by these oil wells. Compared with the oilfield areas, the soil around P sites were more affected by traffic sources that produced to a high proportion of HMW PAHs in the soil. As for the remaining sampling points (I/A/U/B), the ΣPAH and ΣAPAH concentrations were relatively low (average 53.27 ng/g and 12.34 ng/g, respectively), and higher proportions of FLU (9.2–31.7%) and PHE (10.4–26.6%) in ΣPAHs and higher proportion of methylphenanthrene (38.3–73.9%) in ΣAPAHs were observed. FLU and PHE are important tracers for low-temperature combustion sources, such as biomass combustion and coal combustion [16], methylphenanthrene also come from biomass/coal combustion [53], hence, soils in the I/A/U/B areas were affected by both oil production and biomass/coal combustion. In the urban and industrial areas, IcdP (average 15.1%) was the most abundant PAHs, revealing the impact of traffic sources.

In the Yellow River Delta, researchers have studied the influence of oil well density [7] and oil well exploitation history [5] on the concentration of PAH in the soil near the oil well. In this study, we found that oil extraction activities may also cause pollution to distant areas. We found the ΣPAH concentration in soil in the YRD region decreased with distance from the oilfield area (r = 0.66; Figure 3c). This trend was more obvious (r = 0.70) when only 2–3 ring PAHs were considered. The ΣAPAH concentration in soil in the YRD region also decreased with distance from the oilfield area (r = 0.63; Figure 3f). Studabaker, et al. [67] found that ΣPAH levels in lichens decreased with increasing distance from the mining and oil production areas in Canada’s oil sands region. Hsu, et al. [10] suggested that some temperature-dependent PAHs (mainly 3–4 ring PAHs) have the ability to migrate between the atmosphere and other surface media (e.g., surface water or surface soil) due to temperature changes (e.g., seasonal variations). Therefore, the migration of PAHs between the atmosphere and soil may lead to the gradual diffusion of 2–4 ring PAHs from the soil near the O/S/P areas to the soil far away from the oil well (e.g., I/A/U/B areas). This also explains the low ΣPAH concentrations observed in the samples from the industrial areas that are far away from oil wells. Higher-molecular-weight APAHs (such as 1M-PYR and 5M-CHR) show higher proportions in oilfield/suburban/pump unit areas (17.3% and 3.6%) than in I/A/U/B areas (7.7% and 1.6%), indicating that higher-molecular-weight APAHs are more difficult to migrate, which is similar to the parent PAHs. Moreover, according to the classification suggested by MaliszewskaKordybach [68], concentrations of PAHs in soil lower than 200 ng/g, between 200 and 600 ng/g, between 600 and 1000 ng/g, or higher than 1000 ng/g correspond with noncontaminated, weakly contaminated, contaminated, or heavily contaminated, respectively. According to this guideline, 25% of the soils in this study were slightly polluted, and the rest were non-contaminated. The 25% of the slightly polluted points were all located in the O, S, and P areas, revealing oil production has an evident influence on PAHs pollution in the surrounding soils.

### 3.3. Sources of PAHs and APAHs

#### 3.3.1. In Air

**Figure 4:**
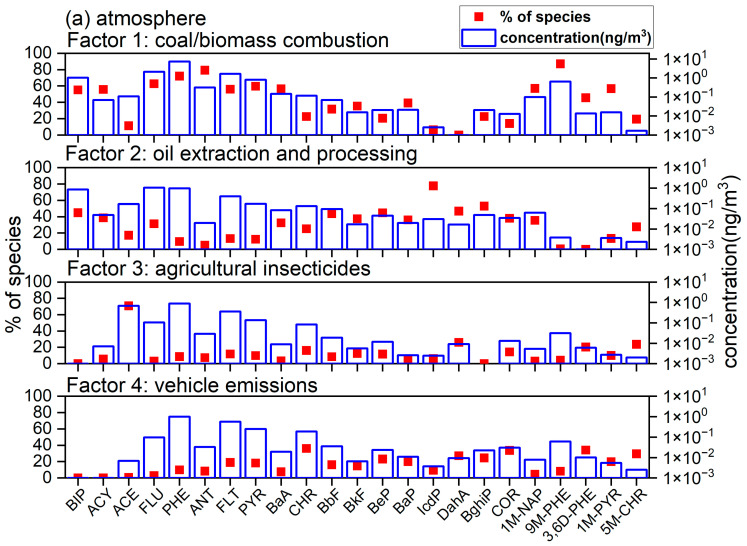

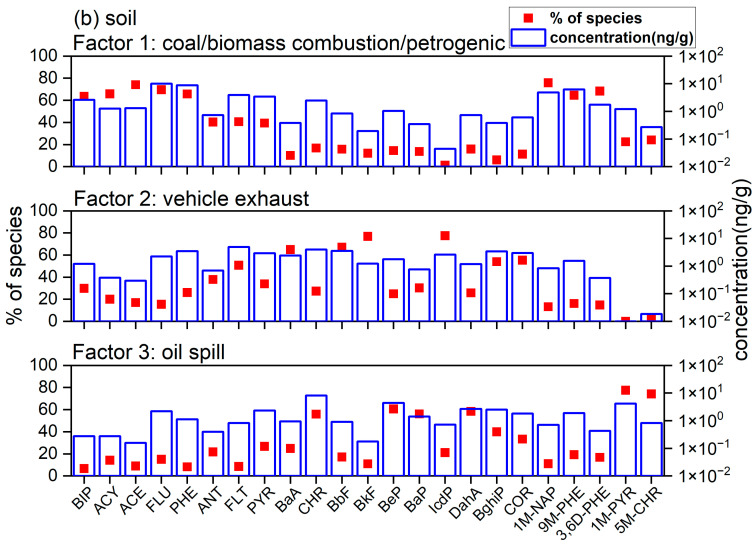
Source profiles of PAHs and APAHs for different sources in the YRD area in (**a**) air and (**b**) soil, resolved by the PMF model.

The EPA PMF 5.0 model was used to identify the sources and corresponding contributions of the PAHs and APAHs in the air and soil in the different functional areas of the YRD. A detailed description of the PMF analysis has been reported in our previous studies [27,31]. As shown in Figure 4a, four factors were resolved for the atmospheric PAHs and APAHs. Factor 1 had high loadings of FLU, PHE, ANT, and 9M-PHE, which agreed well with the emission profile of wood combustion as well as coal combustion [52,69]. Therefore, factor 1 was identified as coal/biomass combustion. Factor 2 exhibited high loadings of IcdP, BghiP, as well as moderate loadings of BIP, ACY, FLU, BeP, BbF and DahA. LMW PAHs, such as BIP and FLU, are important indicators for the volatilization of crude oil and petroleum products [57,70], while ACY, FLU, BaA, and BbF are markers for non-vehicle emission of petroleum combustion [71]. Thus, the source of factor 2 was classified as oil extraction and processing. In factor 3, only ACE accounted for a high loading. It is used to make insecticides and bactericides [61], so factor 3 may represent the source of agricultural insecticides. Factor 4 exhibited high loadings of CHR, COR and moderate loadings of BeP and DahA. CHR is an indicator of diesel emissions [65,72], and DahA and BghiP are the most abundant PAHs in gasoline vehicle emissions [73]. COR is an emission of catalyst and noncatalyst automobiles [74]. Therefore, factor 4 was identified as vehicle emissions.

**Figure 5:**
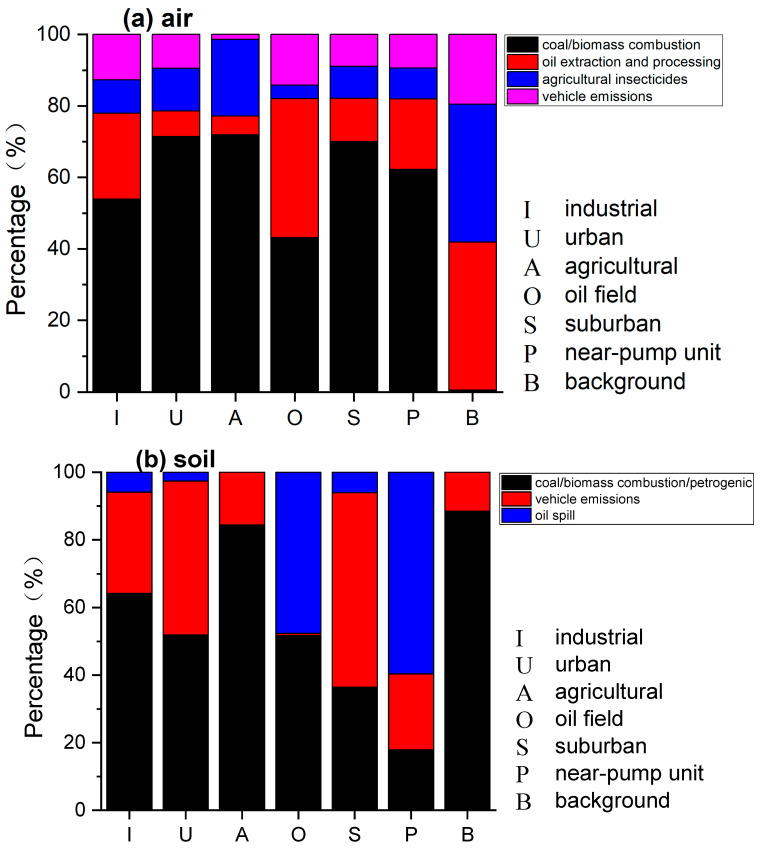
Relative contributions of PAHs and APAHs by different major sources in different functional areas of YRD in (**a**) air and (**b**) soil.

The contributions of atmospheric PAHs and APAHs from the major sources in the different functional areas are presented in Figure 5a. Coal/biomass combustion was the main contributor of atmospheric PAHs for all functional areas (except for the background site), with an average contribution of 62.1%. Dissimilarly, Hsu, et al. [10] found that stack emissions from oil sand mining, upgrading facilities, and emissions from heavy haulers transportation were the main sources of PAHs in the atmosphere in the oil sands area in Canada. Although the proportion of coal in China’s energy structure has decreased from 62.2% in 2016 to 57.7% in 2019, its consumption is on the rise. From 2016 to 2019, coal consumption increased from 3.89 billion tons to 4.02 billion tons (https://data.stats.gov.cn/easyquery.htm?cn=C01&zb=A070R&sj=2021, accessed on 15 June 2023). Along with the acceleration of urbanization in China (from 57.4% in 2016 to 60.6% in 2019), coal consumption for heating has increased rapidly (from 265.7 million tons in 2016 to 344.4 million tons in 2019). In the YRD region, coal/biomass combustion accounted for a large proportion in the urban (71.4%), suburban (70.0%), and agricultural areas (71.9%), as these areas had the greatest demands for heating. Moreover, Hekou District has a larger population (247,595 people in 2010) than Fort McMurray, in Canada (61,374 people in 2011), therefore, the heating demand in the YRD region is greater than in the oil sand areas in Canada. In this study, the contribution of combustion sources in winter were significantly higher than in summer (*p* < 0.01), revealing heating activities in winter had a great contribution to PAHs in the atmosphere [75]. The emissions from oil extraction and processing were the second largest source (17.5%) of atmospheric PAHs in the YRD. These emissions accounted for a high proportion only in the industrial, oilfield, and pump unit areas (average 27.6%), suggesting that the impacts of crude oil production and processing on the atmosphere were mainly concentrated in the areas with the oil extraction industry. As for factor 3 (10.6%), a higher proportion was observed in the agricultural areas (21.4%), reflecting the application of insecticides on farmland and forests. Vehicle emissions (average 9.7%) also contributed notably to oilfield and industrial areas (14.2% and 12.7%, respectively). These areas were the main workplaces for the local industrial population, so daily commuting is common in these two areas. In addition, oil tank trucks were used to transport crude oil from the storage area in the oilfield to the industrial area [8]. These trucks usually use diesel as fuel. Although diesel vehicles account for only about 8% of the total number of motor vehicles in China, their particulate emissions account for 77.8% of the total vehicle emission (https://baijiahao.baidu.com/s?id=1703492584978626064&wfr=spider&for=pc, accessed on 15 June 2023). Additionally, the diesel generators used during the construction of new oil pump units in the oil fields notably contribute to atmospheric PAHs.

#### 3.3.2. In Soil

Three source factors were resolved for soil samples, as shown in Figure 4b. Factor 1 (43.6%) was dominated by a large amount of LMW PAHs and APAHs, such as BIP, ACY, ACE, FLU, PHE, 1M-NAP, 9M-PHE and 3,6D-PHE, and had moderate loadings of ANT, FLT, and PYR. As mentioned in Section 3.3.1, PHE, ANT, FLT, and PYR fit the emission profiles of wood combustion [52] and coal combustion [76]. Furthermore, Szatylowicz and Walendziuk [69] found that ACE was the one of the main component of fly ash samples from coal and wood combustion, and 9-MPH was also reported to be emitted from coal combustion [77]. LMW PAHs and APAHs, e.g., PHE and 1M-NAP, are also typical markers of oil leakage and ground crude oil [2,17]. Thus, factor 1 was identified as mixed source of petrogenic and a combination of coal/biomass combustion. High loadings of Bbf, BkF, IcdP and BaA were found in factor 2 (29.2%). These PAHs species are indicators for gasoline emissions [63,71]. Therefore, factor 2 was determined as the vehicle emissions. In factor 3 (27.2%), CHR, BeP, BaP, DahA, 1M-PYR, and 5M-CHR were the main contributors. and CHR is a marker of oil-well-contaminated soil [23,78]. Additionally, high concentrations of DahA are found in crude oil [62]; therefore, factor 3 was identified as an oil spill.

As shown in Figure 5b, factors 1 and 2 accounted for a large proportion (accounting for 94.0% to 97.4%) in the samples collected in the industrial, urban, and suburban areas, indicating that the PAHs and APAHs in soils in these areas were affected by multiple sources. These areas are densely populated, and traffic is frequent; therefore, coal combustion and vehicle emission activities are common. With the improvement of living standards, the number of motor vehicles in China has risen rapidly from 76.2 million vehicles in 2009 to 261.5 million in 2019, so vehicular emissions have become one of the main sources of PAHs in China [58]. The PAH pollution caused by vehicle exhaust emissions requires additional attention. For the oilfield and near-pump unit areas, factors 1 and 3 were the main factors (accounting for 77.5% to 99.3%), which signifies the impacts of oil extraction and the accompanying oil transportation activities. Wu, et al. [7] studied PAHs in soils with different population densities and oil extraction facility densities in the Shengli Oilfield area and also found that in areas with dense oil fields and relatively sparsely populated areas, crude oil sources are the main source of PAHs in soil, while in areas with moderate to high populations, sources of livelihood and vehicle emissions are the main sources of PAHs in soil. In this study, almost all the PAHs and APAHs in the soil in oilfield and near pump unit areas came from oil field extraction and transportation activities, which is different from the sources for atmospheric PAHs and APAHs in these regions. In the atmosphere, oil extraction sources in the oil field area accounted for 38.9%, while the oil extraction in the near pump area accounted for only 19.8%. For the agricultural areas, factor 1 accounted for a large proportion of 84.4%. This indicates that there may be a large number of coal/biomass combustion sources in these agricultural areas, such as straw incineration. This is consistent with the PMF analysis results for the atmospheric PAHs at agricultural sites. It is worth noting that at the background sites, factor 1 accounted for 88.5% of the PAHs in the soil, and the proportions of LMW and MMW PAHs were relatively high (total 89.9%), especially for FLU (31.2%) and PHE (22.3%). Considering the remote location and sparse population near the background site, the PAHs in the soil may have been caused by atmospheric deposition [66,79]. However, the PMF analysis results of the soil from the background site were inconsistent with that of the atmospheric sample, which needs to be further studied.

### 3.4. Exchange of PAHs/APAHs in Air and Soil

**Figure 6:**
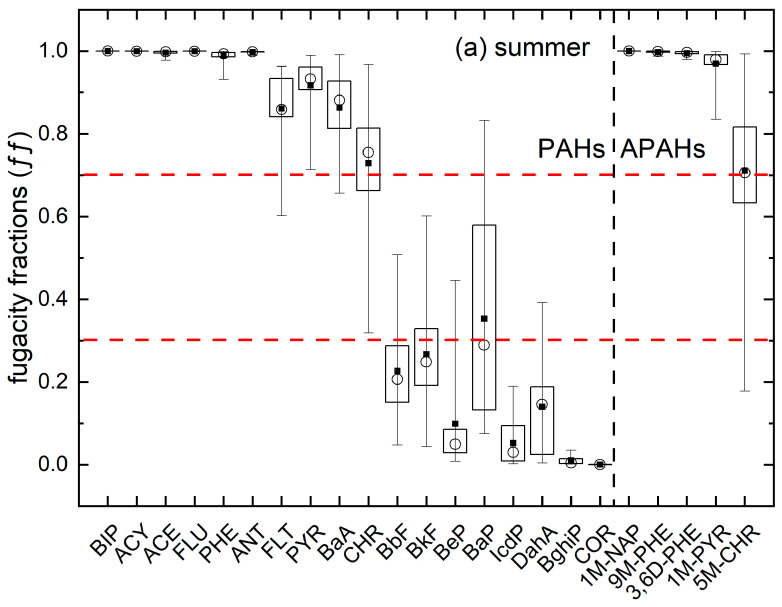

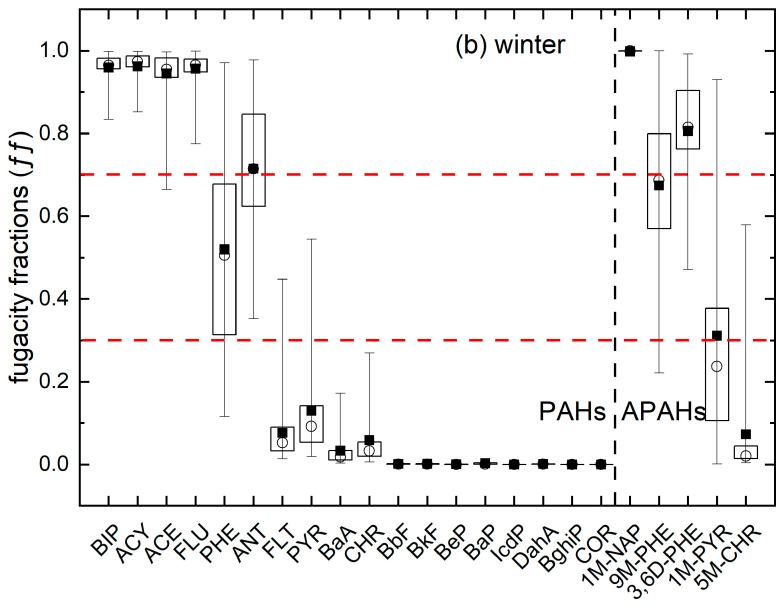
The fugacity fractions (ff) for the air–soil exchange of individual PAHs and APAHs in the YRD in (**a**) summer and (**b**) winter. Note: The lower and upper limits of the whiskers indicate the minimums and maximums, the lower and upper limits of the box indicate 25th and 75th percentiles, the hollow circle in the box indicates median, and the small black square in the box represents mean.

Due to the limited research on PAH in the atmosphere in the Shengli Oilfield area, the exchange trends of PAH and APAH in the atmosphere and soil in this area are still unclear. The $f_{f}$ values of the individual PAHs and APAHs in summer and winter are shown in Figure 6. Most of the $f_{f}$ values were out of the equilibrium range (92.4% in the summer and 90.0% in the winter). In general, the $f_{f}$ value decreased with an increasing number of rings, and all the $f_{f}$ values for the individual PAHs and APAHs in summer were greater than the corresponding values in winter, suggesting that PAHs and APAHs are more likely to volatilize from soil to atmosphere in summer [44], this phenomenon has also been observed in Turkey [21] and is believed to be related to higher temperatures in summer [80]. The $f_{f}$ values for PAHs and APAHs with lower molecular weight (i.e., BIP, ACY, ACE, FLU and 1M-NAP) were greater than 0.7 both in winter and summer, indicating the net volatilization of these substances and suggesting that soil may be a source of these PAHs and APAHs. This trend was probably due to the high volatility of LMW PACs. The average $f_{f}$ value for the PAHs with HMW (i.e., BbF, BkF, BeP, BaP, IcdP, DahA, BghiP, and Cor) were less than 0.3 in both seasons, indicating that these compounds were deposited into the soil from the air (a net “sink” of these chemicals). These deposited HMW PAHs often accumulate in the soil for a long time [44]. The $f_{f}$ values for some 3–4 rings PAHs and APAHs (e.g., PHE, ANT, FLT, PYR, BaA, CHR, 1M-PYR and 5M-CHR) exhibited seasonal differences, suggesting these PACs were more sensitive to seasonal changes than LMW and HMW PAHs and APAHs. These PAHs and APAHs shifted from volatilization in summer to deposition in the winter [81], which may have been caused by changes in rainfall, temperature, and emission profiles [21,44]. These results were consistent with the studies conducted by Wang, et al. [45] and Wang, et al. [44] in other areas of China.

The $f_{f}$ values for the different functional areas are presented in Figure S3**.** The lowest $f_{f}$ values occurred in the urban areas, and $f_{f}$ values from the oilfield sites (except for the background points) were relatively high, suggesting a higher propensity for PAHs and APAHs to volatilize in the atmosphere in oilfield areas. This may partly be related to the greater number of pollution sources in the oilfield areas. For example, oil leakage and ground crude oil will directly pollute the soil. Our results suggest that oil field extraction not only polluted the soil in the oil field area directly but also polluted the soil and atmosphere of the surrounding areas gradually. The fugacity fraction of some PAHs and APAHs (such as FLT, CHR, BbF, BkF, BaP, DahA and 5M-CHR in summer and PHE, ANT, 9M-PHE, 3,6D-PHE, 1M-PYR and 5M-CHR in winter) indicated they were in an equilibrium or volatile state in the oilfield area and in an equilibrium or settlement state in the urban areas. This suggested a potential tendency for those substances to be transported from the oilfield areas to the urban areas.

The urban area has the lowest $f_{f}$ values in this study, Wang, et al. [48] and Wang, et al. [44] found that the $f_{f}$ values in the urban area is higher than those in the suburban areas, which is inconsistent with this study. Dalian is also an oil-producing area, and the main source of PAH in the urban soil is traffic emission/oil spill. However, in the YRD area, the oil extraction facilities are mainly concentrated in the Gudao town and Xianhe town in the suburban area, as well as in the oilfield area. The proportion of oil spill source in the urban soil is limited, which may explain the differences in the distribution trend of air and soil in the urban and suburban regions.

### 3.5. Evaluation of PAHs and APAHs Health Risks

**Figure 7:**
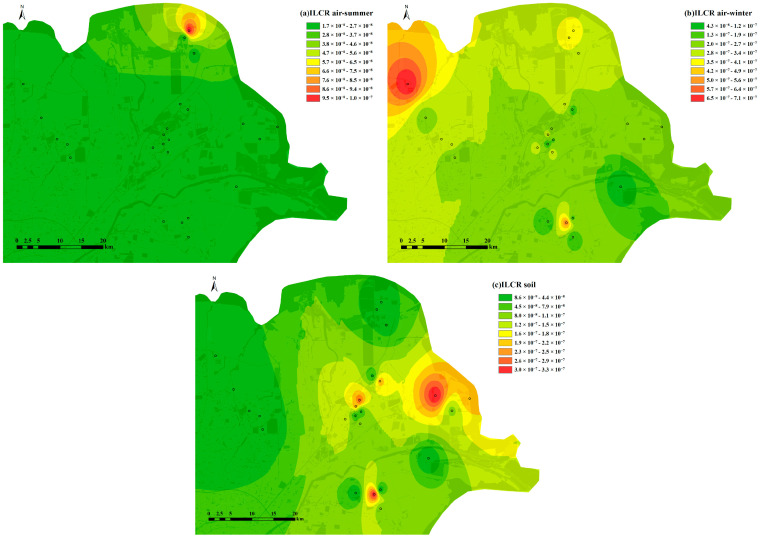
The spatial distributions of the ILCR values for PAHs and APAHs in the YRD area in (**a**) summer air, (**b**) winter air, and (**c**) soil.

The spatial distributions of the total ILCR values for the PAHs and APAHs in air and soil are shown in Figure 7. As for the atmosphere, the total ILCR values in winter ranged from 4.22 × 10^−8^ to 7.11 × 10^−7^, with a mean value of 2.69 × 10^−7^. These were significantly higher (*p* < 0.01) than ILCR values in summer, which ranged from 1.73 × 10^−8^ to 1.04 × 10^−7^, with an average value of 2.42 × 10^−8^. The total ILCR values from the soil samples ranged from 8.22 × 10^−9^ to 3.26 × 10^−7^, with an average of 9.46 × 10^−8^. In both summer and winter, the ILCR values for the atmospheric samples were highest in the industrial areas, followed by pump unit sites and urban areas. BaP (29.7%) was the main contributor to the ILCR in the wintertime atmosphere, while DahA (39.0%) contributed more in summer. BaP mainly comes from combustion sources, such as industrial oil burning, wood combustion, and cooking [65], and DahA in the atmosphere is a characteristic compound for vehicle emissions [27]. Both BaP and DahA have high corresponding TEF values (Both have a TEF value of 1.0). The ILCR values were the highest in the oilfield areas for the soil sample, followed by pump unit sites and the suburban areas. The cancer risk in these regions is about an order of magnitude higher than in other regions. This highlights the health risk for residents and the greater impact of oil extraction activities on soil compared to the atmosphere [11]. The main contributor to the ILCR values in soil was DahA (30.8%), which probably due to vehicle emission and crude oil extraction [27,62]. Generally, the ILCR values for the atmosphere did not exceed the “acceptable” threshold of 10^−6^ set by the US EPA in both seasons [11]. Considering that the passive air sampler method is more effective at collecting gaseous PACs and their derivatives compared to particulate-bounded PACs in the air, the ILCR values calculated in this study are likely to be underestimated. Local residents should be aware of the lifetime cancer risk caused by these air pollutants, especially in winter. The ILCR values for the soil samples from the different functional areas were all below 10^−6^, meaning the cancer risk due to soil pollutants was negligible. This was consistent with a study in an oil sand area in Alberta, Canada [11]. However, according to the predicted carcinogenic risk reported by Wu, et al. [7], if the local oil mining activities, vehicle ownership, and population continue to grow without taking measures to prevent PAHs pollution, the health risks caused by PAHs in soils will increase fourfold by 2040 compared to 2020. If that happens, the ILCR for soil in YRD will exceed 10^−6^, so the ILCR for soil in the YRD region should be closely monitored in the future.

The MEQ results for PAHs and APAHs in air and soil are shown in Table S6. In the atmosphere, the highest MEQ values occurs in industrial areas followed by pump unit sites in both summer and winter, and in the soil, the MEQ shows higher values in pump unit sites followed by oilfield areas and suburban areas—these areas are all close to oil extraction facilities. In both the atmosphere and soil, the MEQ value near the pump unit site were relatively high, which is similar to ILCR values, indicating that oil extraction facilities located near residential areas not only increase the risk of cancer but also increase the risk of mutation to local residents.

## 4. Conclusions

The concentrations, sources, air–soil exchange, and health risks of 18 PAHs and 5 APAHs were studied through comprehensive analyses of air and soil samples collected in the YRD region in North China. The concentrations of atmospheric ΣPAHs ranged from 2.26 to 135.83 ng/m^3^, while the ΣAPAHs in the atmosphere ranged from 0.04 to 16.31 ng/m^3^. Both ΣPAH and ΣAPAH concentration in winter were significantly higher than those in summer, which was mainly due to the influence of residential heating. The total ΣPAH and ΣAPAH concentrations in the soil samples were within the range of 33.96–408.94 ng/g and 6.39–211.86 ng/g, respectively. Compared with previously reported data, the concentration of soil PAHs in the YRD was relatively low in China but higher than in the oil sand region in Canada. PHE accounted for a considerable proportion (40.3%) of atmospheric PAHs, while 9M-PHE accounting for 60.5% of atmospheric APAHs. Higher ΣPAH and ΣAPAH concentrations in the atmosphere occurred in the densely populated urban, industrial, and suburban areas. The dominant PAHs in the soil samples were FLU (14.4%), PHE (14.2%), and CHR (9.4%), while 1M-NAP (35.9%) and 9M-PHE (37.6%) were the dominant APAHs, and higher ΣPAH and ΣAPAH concentrations in soil were found in the oilfield, suburban, and pump unit areas. These results indicate that there were different sources for the atmospheric and soil PAHs and APAHs. PMF analysis revealed that the main sources for atmospheric PAHs and APAHs were coal/biomass combustion sources, whereas coal/biomass combustion sources, oil spills, and vehicle exhaust were the main contributors to PAHs and APAHs in the soil. The fugacity ratios suggested that soil was a source of LMW PAHs and APAHs, and was a sink for HMW PAHs. Furthermore, 3–4 rings PAHs and APAHs exhibited distinct patterns between summer and winter, which is attributable to the temperature and seasonal emission variances. The urban sites had lower $f_{f}$ values, and samples from the oilfield exhibited higher $f_{f}$ values. This indicates a potential tendency for PAHs and APAHs to be transported from oilfield areas to urban areas. The main contributors to the ILCR values for the air samples in summer and winter were DahA (39.0%) and BaP (29.7%), respectively. Additionally, DahA (30.8%) was a significant contributor to the ILCR values in soil. The ILCR of the Σ(PAHs+APAHs) in the air and soil were negligible since the ILCR values were below the risk threshold of 10^−6^.

## Data Availability

Not applicable.

## Supplementary Materials

The following supporting information can be downloaded at: https://www.mdpi.com/article/10.3390/toxics11060540/s1, Table S1. Details for the sampling sites and sampling information. Table S2. Abbreviation, method detection limit for PAHs and APAHs in air (ng/m_3_) and soil (ng/g). Table S3. Toxic equivalent factor (TEF) for individual PACs. Table S4. Incremental lifetime cancer risk assessment exposure parameters. Table S5. Temperature dependent LogKoa of different PAHs and APAHs. Table S6. Mean values of MEQ in air and soil samples in different functional areas. Figure S1. The concentrations of individual PAHs at 24 sampling sites, (a) summer air(ng/m_3_); (b) winter air(ng/m_3_); (c) soil(ng/g). Figure S2. The concentrations of individual APAHs at 24 sampling sites, (a) summer air(ng/m_3_); (b) winter air(ng/m_3_); (c) soil(ng/g). Figure S3. Fugacity fractions (ff) for individual PAHs and APAHs in industrial (I), urban (U), agricultural (A), oil field (O), suburb (S), pump unit (P) and background (B) areas in (a)summer and (b) winter.
