# Supplementary material for: Spatial Distribution, Sources, Air–Soil Exchange, and Health Risks of Parent PAHs and Derivative-Alkylated PAHs in Different Functional Areas of an Oilfield Area in the Yellow River Delta, North China
Source: Toxics. 2023 Jun 17;11(6):540. doi: 10.3390/toxics11060540 (PMC10302137; doi:10.3390/toxics11060540)
Supplement: Supplementary file 1 [file toxics-11-00540-s001.zip › toxics-2397818-supplementary.pdf]

Supplementary material

Spatial distribution, Sources, Air-Soil Exchange, and Health Risks of Parent PAHs and Derivative-Alkylated PAHs in Different Functional Areas of an Oilfield Area in the Yellow River Delta, North China

Xiongfei Zhang [a], Anan Qi [a], Pengcheng Wang [a], Qi Huang [a], Tong Zhao[a], Caiqing Yan [a], Lingxiao Yang [a,b,*] and Wenxing Wang [a]

Captions of Tables and Figures

Table S1. Details for the sampling sites and sampling information.

Table S2. Abbreviation, and method detection limit for PAHs and APAHs in air (ng/m3) and soil (ng/g).

Table S3. Toxic equivalent factor (TEF) for individual PACs

Table S4. Incremental lifetime cancer risk assessment exposure parameters.

Table S5. Temperature dependent LogKoa of different PAHs and APAHs.

Table S6. Mean values of MEQ in air and soil samples in different functional areas.

Figure S1. The concentrations of individual PAHs at 24 sampling sites, (a) summer air(ng/m^3); (b) winter air(ng/m^3); (c) soil(ng/g).

Figure S2. The concentrations of individual APAHs at 24 sampling sites, (a) summer air(ng/m^3); (b) winter air(ng/m^3); (c) soil(ng/g).

Figure S3. Fugacity fractions (ff) for individual PAHs and APAHs in industrial (I), urban (U), agricultural (A), oil field (O), suburb (S), pump unit (P) and background (B) areas in (a)summer and (b) winter.

Table S1. Details for the sampling sites and sampling information.

Functional Type	Site	Longitude(E)	Latitude(N)	Days Deployed (Summer/Winter)
Industrial	I1	118°26′2″	37°59′33″	60/60
	I2	118°28′48″	37°55′17″	60/60
	I3	118°52′27″	38°5′53″	60/60
	I4	118°51′43″	38°4′59″	60/60
	I5	118°53′10″	38°3′0″	60/60
Urban	U1	118°31′14″	37°52′34″	60/60
	U2	118°32′52″	37°51′53″	60/60
	U3	118°33′18″	37°50′13″	60/60
Agricultural	A1	118°51′30″	37°42′23″	60/60
	A2	118°47′49″	37°42′2″	60/60
	A3	118°51′44″	37°39′59″	60/60
Oilfield	O1	119°1′3″	37°54′53″	60/60
	O2	119°6′7″	37°53′35″	60/60

	O3	119°3′17″	37°52′6″	60/60
Suburban	S1	118°50′49″	37°56′41″	60/60
	S2	118°52′1″	37°55′59″	60/60
	S3	118°48′54″	37°52′13″	60/60
	S4	118°48′0″	37°51′44″	60/60
	S5	118°50′44″	37°41′49″	60/60
Near Pump Unit	P1	118°48′41″	37°53′38″	60/60
	P2	118°46′23″	37°51′18″	60/60
	P3	118°48′5″	37°52′54″	60/60
	P4	118°48′43″	37°50′42″	60/60
Background	B	118°58′44″	37°46′35″	60/60

Table S2. Abbreviation, and method detection limit for PAHs and APAHs in air (ng/m^3) and soil (ng/g).

Compound	Abbreviations	Ring	MDL-Air (ng/m^3)	MDL-Soil (ng/g)
PAHs				
Biphenyl	BIP	2	0.002	0.03
Acenaphthylene	ACY	3	0.001	0.03
Acenaphthene	ACE	3	0.005	0.10
Fluorene	FLU	3	0.002	0.03
Phenanthrene	PHE	3	0.001	0.02
Anthracene	ANT	3	0.005	0.10
Fluoranthene	FLT	4	0.005	0.10
Pyrene	PYR	4	0.005	0.10
Benzo(a)anthracene	BaA	4	0.011	0.20
Chrysene	CHR	4	0.011	0.20
Benzo(b)fluoranthene	BbF	5	0.029	0.50
Benzo(k)fluoranthene	BkF	5	0.029	0.50
Benzo(e)pyrene	BeP	5	0.015	0.25
Benzo(a)pyrene	BaP	5	0.029	0.50
Indeno(1,2,3-cd)pyrene	IcdP	5	0.049	0.80
Dibenzo(a,h)anthracene	DahA	6	0.061	1.00
Benzo(g,h,i)perylene	BghiP	6	0.049	0.80
Coronene	COR	7	0.125	2.00
APAHs				
1-Methylphenanthalene	1M-NAP		0.002	0.05
9-Methylphenanthrene	9M-PHE		0.027	0.50
3,6-Dimthylphenanthrene	3,6D-PHE		0.011	0.20
1-Methylpyrene	1M-PYR		0.006	0.10
5-Methylchrysene	5M-CHR		0.012	0.20

Table S3. Toxic equivalent factor (TEF) for corresponding individual PACs

Compound	TEF
ACY	0.0010
ACE	0.0010
FLU	0.0010
PHE	0.0010
ANT	0.0100
FLT	0.0010
PYR	0.0010
BaA	0.1000
CHR	0.0100
BbF	0.1000
BkF	0.1000
BaP	1.0000
IcdP	0.1000
DahA	1.0000
BghiP	0.0100
1-MNAP	0.0025
1-MPYR	0.1000
5-MCHR	1.0000

Table S4. Parameters for the incremental lifetime cancer risk assessment.

Exposure Parameter	Units	Value	References
Carcinogenic Slope Factor of Inhalation ($CSF_{inhalation}$)	$(mg\ kg^{-1}\ day^{-1})^{-1}$	3.85	(Hu et al., 2007; Peng et al., 2011)
Carcinogenic Slope Factor of Ingestion ($CSF_{ingestion}$)	$(mg\ kg^{-1}\ day^{-1})^{-1}$	7.3	(Hu et al., 2007; Peng et al., 2011)
Carcinogenic Slope Factor of Dermal (CSF_{dermal})	$(mg\ kg^{-1}\ day^{-1})^{-1}$	25	(Hu et al., 2007; Peng et al., 2011)
Inhalation Rate ($IR_{inhalation}$)	m^3/day	20	(Ali, 2019)
Ingestion Rate (IR_{ingest})	mg/day	30	(USEPA, 2011)
Exposure Frequency (EF)	day/year	350	(Hu et al., 2007; Ali, 2019)
Exposure Duration (ED)	year	24	(USEPA, 2002)
Body Weight (BW)	kg	70	(USEPA, 2002)
Average Lifespan of Human (AT)	day	25550	(Wang et al., 2011)
Skin Area (SA)	cm^2/day	5700	(BMEPRI, 2007; Peng et al., 2011)
Skin Adherence Factor (SAF)	mg/cm^2	0.07	(BMEPRI, 2007; Peng, et al., 2011)
Dermal Absorption Factor (ABS)	-	0.13	(USEPA, 2002)
Particle Emission Factor (PEF)	m^3/kg	1.36×10^9	(USEPA, 2002)

Table S5. Temperature dependent LogKoa of different PAHs and APAHs.

Compound	A	B	Summer (T=292.16)	Winter (T=270.29)
BIP	-1.85	2313	6.07	6.71
ACY	-2.41	2681	6.77	7.51
ACE	-2.00	2409	6.25	6.92
FLU	-2.08	2462	6.35	7.03
PHE	-2.76	2918	7.22	8.03
ANT	-2.76	2918	7.22	8.03
FLT	-3.88	3661	8.64	9.66
PYR	-3.88	3661	8.64	9.66
BaA	-4.55	4099	9.48	10.62
CHR	-4.55	4099	9.48	10.62
BbF	-5.68	4847	10.92	12.26
BkF	-5.68	4847	10.92	12.26
BeP	-6.30	5259	11.70	13.16
BaP	-5.68	4847	10.92	12.26
IcdP	-6.80	5595	12.35	13.90
DahA	-6.34	5285	11.75	13.22
BghiP	-7.28	5912	12.95	14.59
COR	-8.22	6532	14.14	15.95
1M-NAP	-1.55	2116	5.69	6.28
9M-PHE	-3.17	3187	7.74	8.62
3,6D-PHE	-3.59	3463	8.27	9.22
1M-PYR	-4.30	3936	9.17	10.26
5M-CHR	-4.96	4374	10.01	11.22

Notes: LogKoa=A+B/T; The KOA of PAH and APAHs were obtained from(Harner, 2021), otherwise estimated by EPI-suite.

Table S6. Mean values of MEQ in air and soil samples in different functional areas.

Functional area	Summer air (ng/m^3)	Winter air (ng/m^3)	Soil (ng/g)
Industrial	0.094852	1.004387	3.950608
Urban	0.057963	0.748245	4.206544
Agricultural	0.044542	0.374574	3.906807
Oilfield	0.050779	0.821753	19.2451
Suburban	0.046505	0.611221	18.74586
Pump unit	0.060128	0.904332	21.28262
Background	0.043384	0.119419	1.562069

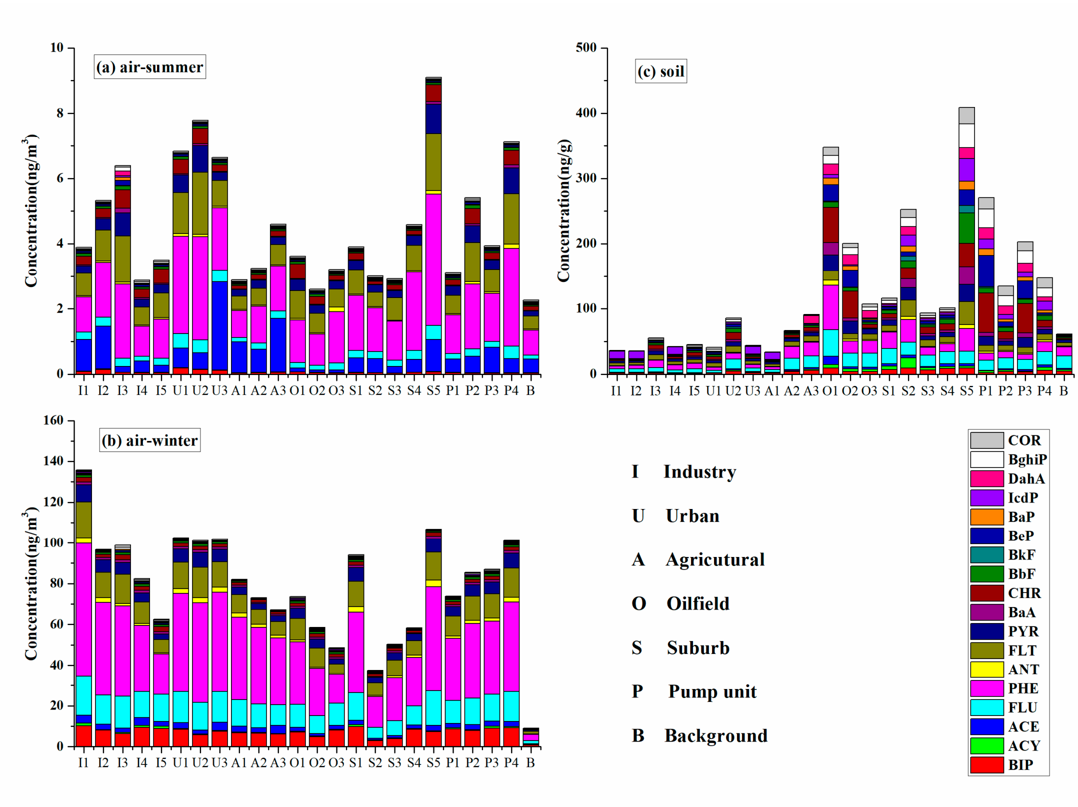

Figure S1. The concentrations of individual PAHs at the 24 sampling sites in (a) summer air (ng/m^3); (b) winter air (ng/m^3); and (c) soil (ng/g).

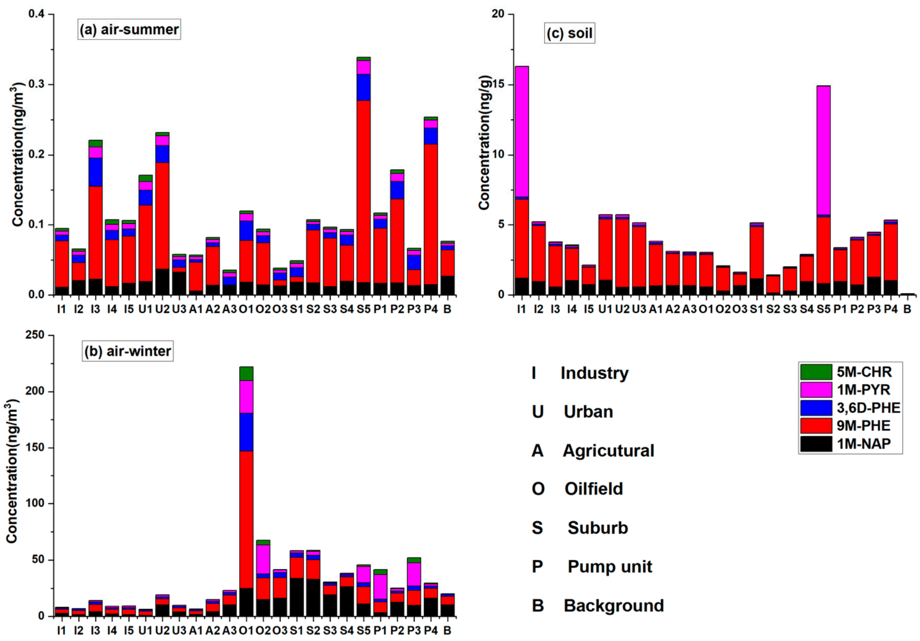

Figure S2. The concentrations of individual APAHs at the 24 sampling sites in (a) summer air (ng/m^3); (b) winter air (ng/m^3); and (c) soil (ng/g).

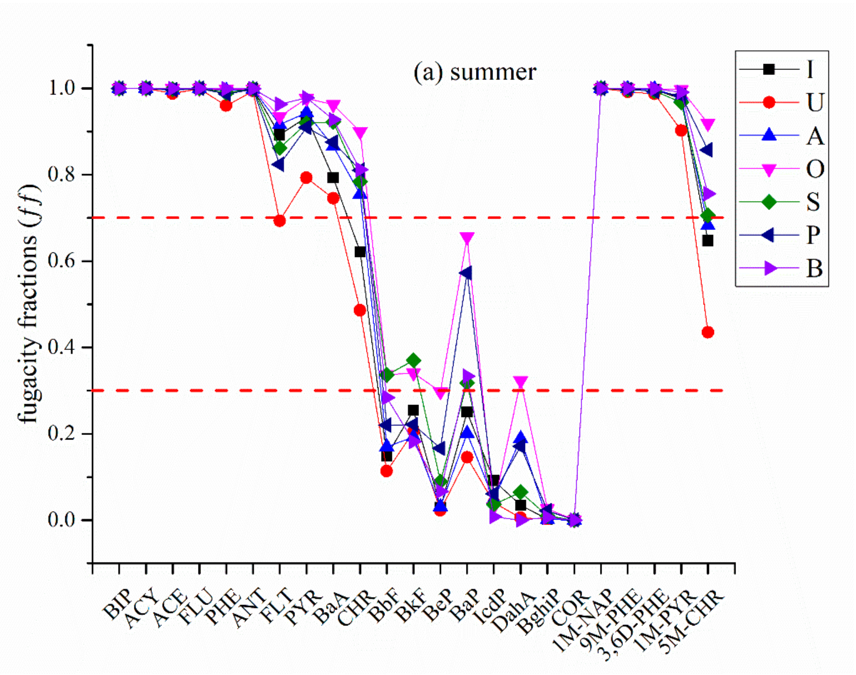

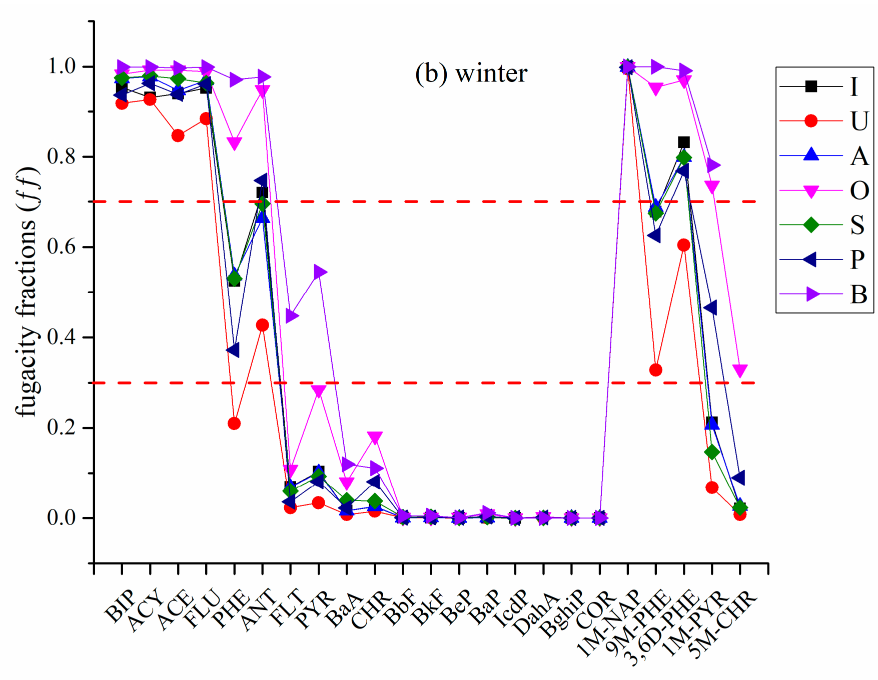

Figure S3. Fugacity fractions (ff) for individual PAHs and APAHs in industrial (I), urban (U), agricultural (A), oil field (O), suburb (S), pump unit (P) and background (B) areas in (a)summer and (b) winter.

References

Ali, N., 2019. Polycyclic aromatic hydrocarbons (PAHs) in indoor air and dust samples of different Saudi microenvironments; health and carcinogenic risk assessment for the general population. Science of The Total Environment 696.

Hu, Y.D.; Bai, Z.P.; Zhang, L.W.; Wang, X.; Zhang, L.; Yu, Q.C.; Zhu, T. Health risk assessment for traffic policemen exposed to polycyclic aromatic hydrocarbons (pahs) in tianjin, china. *Sci. Total Environ.* **2007**, *382*, 240–250.

Harner, T., 2021. 2021 v10 Template for calculating PUF and SIP disk sample air volumes April28.

Peng, C.; Chen, W.P.; Liao, X.L.; Wang, M.E.; Ouyang, Z.Y.; Jiao, W.T.; Bai, Y. Polycyclic aromatic hydrocarbons in urban soils of beijing: Status, sources, distribution and potential risk. *Environ. Pollut.* **2011**, *159*, 802–808.

USEPA, 2002. Supplemental guidance for developing soil screening levels for superfund sites. Office of Emergency and Remedial Response, Washington DC.

USEPA, 2011. Exposure Factors Handbook: 2011 Edition (Chapter 5-Soil and Dust Ingestion). U.S. Environmental Protection Agency, Washington DC.
